# Long-term fasting: Multi-system adaptations in humans (GENESIS) study–A single-arm interventional trial

**DOI:** 10.3389/fnut.2022.951000

**Published:** 2022-11-17

**Authors:** Franziska Grundler, Magalie Viallon, Robin Mesnage, Massimiliano Ruscica, Clemens von Schacky, Frank Madeo, Sebastian J. Hofer, Sarah J. Mitchell, Pierre Croisille, Françoise Wilhelmi de Toledo

**Affiliations:** ^1^Buchinger Wilhelmi Clinic, Überlingen, Germany; ^2^UJM-Saint-Etienne, INSA, CNRS UMR 5520, INSERM U1206, CREATIS, F-42023, Université de Lyon, Saint-Étienne, France; ^3^Department of Radiology, University Hospital Saint-Étienne, Saint-Étienne, France; ^4^Department of Medical and Molecular Genetics, Faculty of Life Sciences and Medicine, King’s College London, London, United Kingdom; ^5^Department of Pharmacological and Biomolecular Sciences, Università degli Studi di Milano, Milan, Italy; ^6^Omegametrix, Martinsried, Germany; ^7^Institute of Molecular Biosciences, NAWI Graz, University of Graz, Graz, Austria; ^8^BioHealth Graz, Graz, Austria; ^9^BioTechMed Graz, Graz, Austria; ^10^Department of Health Sciences and Technology, ETH Zürich, Schwerzenbach, Switzerland

**Keywords:** organ size, lipoprotein metabolism, metabolomics, microbiome, long-term fasting, magnetic resonance imaging (MRI), protein utilisation

## Abstract

Fasting provokes fundamental changes in the activation of metabolic and signaling pathways leading to longer and healthier lifespans in animal models. Although the involvement of different metabolites in fueling human fasting metabolism is well known, the contribution of tissues and organs to their supply remains partly unclear. Also, changes in organ volume and composition remain relatively unexplored. Thus, processes involved in remodeling tissues during fasting and food reintroduction need to be better understood. Therefore, this study will apply state-of-the-art techniques to investigate the effects of long-term fasting (LF) and food reintroduction in humans by a multi-systemic approach focusing on changes in body composition, organ and tissue volume, lipid transport and storage, sources of protein utilization, blood metabolites, and gut microbiome profiles in a single cohort. This is a prospective, single-arm, monocentric trial. One hundred subjects will be recruited and undergo 9 ± 3 day-long fasting periods (250 kcal/day). We will assess changes in the composition of organs, bones and blood lipid profiles before and after fasting, as well as high-density lipoprotein (HDL) transport and storage, untargeted metabolomics of peripheral blood mononuclear cells (PBMCs), protein persulfidation and shotgun metagenomics of the gut microbiome. The first 32 subjects, fasting for 12 days, will be examined in more detail by magnetic resonance imaging (MRI) and spectroscopy to provide quantitative information on changes in organ volume and function, followed by an additional follow-up examination after 1 and 4 months. The study protocol was approved by the ethics board of the State Medical Chamber of Baden-Württemberg on 26.07.2021 and registered at ClinicalTrials.gov (NCT05031598). The results will be disseminated through peer-reviewed publications, international conferences and social media.

**Clinical trial registration:**

[ClinicalTrials.gov], identifier [NCT05031598].

## Introduction

The health-promoting effects of long-term fasting (LF), lasting more than 4 days and up to several weeks, are increasingly documented (1–3). Our group has shown that LF improves cardiovascular (CV) risk factors such as hypertension even in medicated subjects, lipoprotein distribution, non-alcoholic fatty liver symptoms, inflammatory parameters, oxidative stress, and gut microbiota profiles (4–9). A large observational study of 1,422 subjects underlined the safety, tolerability, and therapeutic efficacy of LF from 4 to 21 days (10). However, the precise physiological consequences of acute LF periods and subsequent food reintroduction phases are still not comprehensively understood.

A key molecular mechanism of fasting-mediated health benefits is the metabolic switch from glucose, as the main energy source, to the utilization of fat-derived lipids and ketones (11). During the first 48 fasting hours, the initial glycogen depletion is accompanied by fat and–to a lesser extent–protein utilization. The proportion of protein usage then decreases after the activation of protein-sparing mechanisms (12). In the 1980s, concerns about protein loss–and subsequent muscle decline–were raised for zero-calorie diets lasting more than 100 days, based on nitrogen balance measurements (13, 14), while the origin of protein breakdown was not determined. Conversely, in a recent study investigating 10 days of fasting, including daily moderate physical activity in 16 healthy men, muscle strength (grip and leg strength) was observed to be maintained and even increased (15).

Due to substrate mobilization in several metabolically active tissues, the size and weights of organs changes. Liver, spleen, kidneys and skeletal muscle mass decrease in rats after long periods of severe 50% calorie restriction (CR), while the brain and testes seem unaffected (16). Multiple cycles of fasting-mimicking-diet (FMD; 10% of normal daily calorie intake) in mice restored insulin-generating β-cells, promoted stress resistance, self-renewal division of stem cells, and hematopoietic lineage-balanced regeneration (17–19). Few clinical studies of long-term CR are available in humans. A 12-week semi-starvation intervention in 32 men reduced the heart’s overall size proportionally to the body weight and showed a trend of recovery after food reintroduction (20). Fat loss plays a role in the shrinkage of organ volume. After a 10-day fast in men, enhanced lipid oxidation was shown in muscles (15).

Furthermore, fasting-induced autophagy and apoptosis are also thought to contribute to organ shrinkage (21). Decreased protein content during fasting is restored upon food reintroduction, as documented by the switch to a positive nitrogen balance (22, 23). Consequently, organ size can be rebuilt due to *de novo* synthesis and stem cell activation, possibly conferring beneficial effects on tissue functionality. To the best of our knowledge, no extensive human studies exist that systematically analyzed the body composition after a LF period using state-of-the-art magnetic resonance imaging/spectroscopy (MRI/MRS) approaches. One case report outlined the effects of a 14-day fasting period in a healthy man by MRI/MRS, showing changes in adipose tissue distribution and fatty acid composition in multiple organs (24).

Thus, in addition to body and organ composition, we will investigate lipid metabolism during LF. Serum triglycerides, low-density-lipoprotein (LDL) and atherogenic subfractions (e.g., small dense LDL) significantly decreased after 2 weeks of fasting, pointing to a reduced lipid-associated atherogenic risk (9). Of note, high-density lipoprotein cholesterol (HDL-C) is inversely associated with both CV disease and mortality (25) and pharmacological interventions aiming at raising HDL-C levels were not successful thus far (26, 27). Hence, there is an increasing interest in measuring high-density lipoprotein (HDL) function, as reflected by cholesterol efflux capacity (CEC) and the serum cholesterol loading capacity (CLC), which are both indices of CV risk (28, 29). This study will investigate whether LF influences the reverse transport of cholesterol from the periphery to the liver. In addition, erythrocyte membrane fatty acid composition reliably predicts total mortality and clinical events (30–32). Therefore, we will determine the fatty acid composition of erythrocytes and the omega-3/omega-6 ratio during LF.

The exact cascade of molecular mechanisms leading to therapeutic benefits of different fasting regimes remains elusive, at least in humans (33). Interestingly, organisms maintain cellular and organismal homeostasis during fasting, *inter alia*, by well-orchestrated biochemical and (epi) genetic mechanisms (11, 34). The progress of precision metabolomics technologies recently opened new avenues to study the changing metabolic landscape during the fasting state in a spatiotemporal manner (35). Thus, this study will shed light on the intracellular metabolic consequences of LF in peripheral blood mononuclear cells (PBMCs), focusing on lipids and polyamines, which are essential for diverse cellular functions (36). Another aspect of LF-induced metabolic effects is increased endogenous hydrogen sulfide (H2S) production, a physiological gasotransmitter (37, 38) *via* sulfhydration/persulfidation (39) with health-promoting properties (REF). In mice, 40% dietary restriction increased H2S levels, which is required for the geroprotective effects of CR (37). Thus, we will investigate the impact of LF on H2S-mediated signaling in humans.

Finally, we will explore gut microbiota changes during LF. LF does not eliminate the gut microbiota, but elicits profound changes in its composition (6, 40). In our recent study, changes in bacteria profiles caused by fasting were associated with serum glucose and fecal branched-chain amino acids (6), suggesting that the gut microbiota can influence fasting-induced changes in energy metabolism. A more recent study even identified a bacteria which abundance correlates with serum concentrations of 3-hydroxybutyrate (41).

**FIGURE 1:**
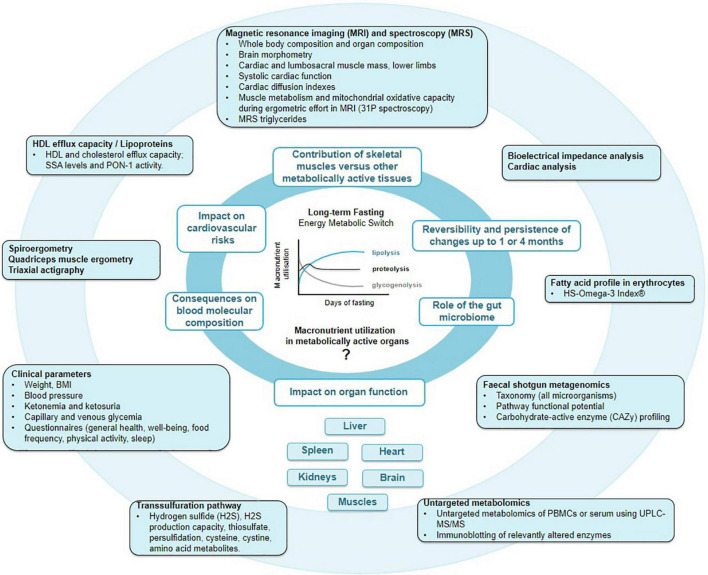
Main outcomes of the GENESIS study. By pooling knowledge acquired by macroscopic multi-organ fat distribution and composition provided by magnetic resonance imaging (MRI), with erythrocyte fatty acids, lipoprotein, peripheral blood mononuclear cell (PBMC) metabolome, protein persulfidation, and gut microbiome profiles, we aim to gain a deeper comprehension of molecular and physiological changes during fasting and food reintroduction.

In light of these incognita, we designed a multi-stage single-arm interventional trial to apply state-of-the-art methods to elucidate the fasting-induced changes in metabolism, body composition, organ size and function, and the gut microbiome (Figure 1).

## Methods and analysis

### Aims

First, the GENESIS study aims to document changes in body composition and the contribution of the main metabolically active tissues (skeletal muscular tissues, adipose tissues, liver, heart, spleen, kidneys, and brain) to the metabolic switch during a 12-day fasting period. Changes in the size and mass of organs will be quantified. We hypothesize that a re-expansion and regeneration will follow a transient decrease of organ volume during fasting up to 4 months after food reintroduction. Protein resynthesis upon food reintroduction might correspond to an acceleration of the physiological protein turnover, which will be evaluated by measuring nitrogen excretion. Additionally, the organs’ specific compositions, especially the fat components, and their function will be documented along the fasting process. We hypothesize that these multi-system changes are safe, and that they could also be reflected by an improved cardiac and skeletal muscle metabolism and mitochondrial oxidative capacity which will be investigated by spiroergometry.

Second, the study focuses on fat storage, function, and exchange in adipose tissue, liver, spleen, kidney, and splanchnic tissue. Among the lipids studied, we focus on cholesterol metabolism during fasting, particularly sub-types of HDL, but also CEC and fatty acid profiles in the erythrocyte membrane.

Third, we will measure PBMC metabolome profiles to reveal cellular metabolic changes in circulating, easily accessible immune cells.

Fourth, we will determine whether persulfidation, which contributes to the maintenance of cellular oxidative functions, changes during fasting.

Last, we will measure if various metabolic and physiological changes, as described above, correlate to individual gut microbiota profiles. The composition and function of the fecal microbiota will be evaluated during LF using shotgun metagenomics. This will help us understand how intestinal microorganisms are linked to human physiology during fasting.

### Study design

**FIGURE 2:**
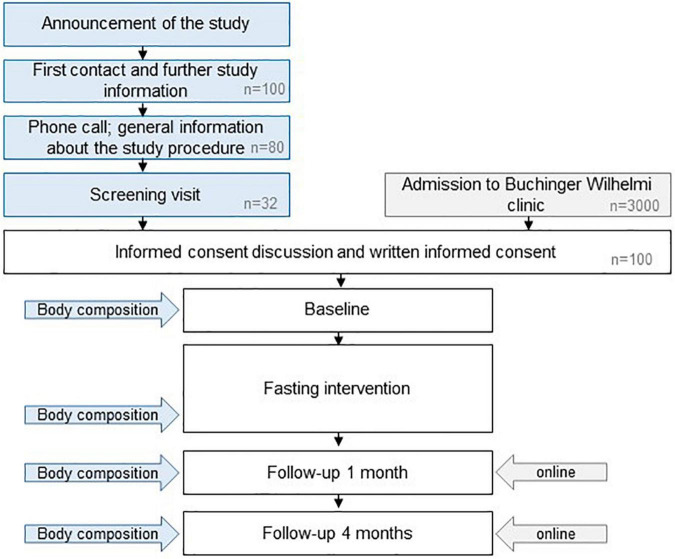
Study design. Specific sessions for the subjects enrolled in the first stage of the projects are indicated in blue. The estimated numbers of volunteers needed to recruit the sample size of 100 subjects are indicated in gray.

The GENESIS study is a prospective, monocentric, single-arm interventional study using a two-stage design. A total of 100 subjects will be included (Figure 2). The first 32 participants will undergo an augmented examination plan, including MRI/MRS scans to evaluate body composition changes at four time points: prior to and at the end of 12 fasting days, 1 and 4 months post fasting. This sub-cohort is statistically powered for detecting changes in whole body composition. Sixty-eight additional subjects will then be included for gut microbiota and lipid profile analyses. The study follows the STROBE guidelines (42).

### Recruitment

The study site is a specialized center for long-term therapeutic fasting under medical supervision. Detailed information about the GENESIS study will be provided orally and in a written manner to potential participants. For the recruitment of the first phase (32 participants), participation calls will be distributed in the study center, on social media channels of the Buchinger Wilhelmi clinic and in institutions working on fasting and nutrition. Informed consent will be collected prior to the start of the fasting period and any baseline measurements. Since participants of the first stage need to be physically present at four time points (prior and post fasting, follow-up visits after 1 and 4 months), they will stay at the Buchinger Wilhelmi clinic without charges. The remaining 68 participants will be recruited prospectively during 1 year from the regular pool of customers voluntarily undergoing fasting in the Buchinger Wilhelmi clinic in Überlingen. These participants will not receive any financial incentives. The recruitment started in September 2021. Thus, the last follow-up will be prospectively performed in January 2023. Study participants will be informed by email about their results and publications based on data collected in the GENESIS trial.

### Participants

Men and women aged 20–75 years with BMIs between 22 and 35 kg/m^2^ are eligible to participate if they match further eligibility criteria, as listed in Table 1. Any contraindication to fasting, as defined in the guidelines of fasting therapy including kidney, liver or cerebrovascular insufficiency (43) automatically leads to exclusion. Fulfilment of additional criteria related to contraindications for the MRI/MRS scans is required for participants of the MRI subgroup (Table 1).

**TABLE 1:** Eligibility criteria.

Inclusion criteria	Exclusion criteria
• Men and women • Age between 20–75 years • BMI between 22–35 kg/m^2^ • Negative COVID-19 test • Available written declaration of consent	• Intake of medication (cardiovascular diseases, lipid, and glucose metabolism) • Chronic manifest psychical and psychiatric diseases • Participation in another study • Pregnancy or breastfeeding • Active uncontrolled gastrointestinal disorders including ulcerative colitis, Crohn’s disease, indeterminate colitis, severe irritable bowel syndrome, persistent infectious gastroenteritis, persistent or chronic diarrhea of unknown etiology, and recurrent *Clostridium difficile* infection • Major surgery of the gastrointestinal tract, in the past 5 years. Any major bowel resection at any time • Intake of antibiotics in the last 2 months • In the MRI subgroup, any MRI contraindication (claustrophobia, pacemakers, MR-incompatible prosthetic valves, metallic implants, and foreign metallic body)

### Fasting intervention

The subjects will receive a plant-based, organic calorie-restricted diet (600 kcal/day) 1 day before the study begins. The 12-day fasting period will be initiated by emptying the intestinal tract *via* the intake of a laxative [e.g., sodium sulfate (Glaubers’ Salt) in the morning or sodium picosulfate (Laxoberal^®^, Sanofi-Aventis, Germany) the night before]. During the fasting period, the energy intake is limited to 200–250 kcal/day (10), which will be achieved with 0.25 L freshly squeezed, organic fruit juice at noon, 0.25 L vegetable soup in the evening and 20 g honey. Participants will be asked to drink a minimum of 2 L of water or non-caloric, caffeine-free herbal teas that are purchased by certificated, organic companies (SONNENTOR Kräuterhandelsgesellschaft mbH, Sprögnitz, Austria; Ulrich Walter GmbH, Diepholz, Germany). Every second fasting day, the colon will be emptied by an enema or a laxative. On the last fasting day, plant-based organic food will be progressively reintroduced over 3–4 consecutive days (800–1,600 kcal/day).

Medical doctors and nurses will supervise the fasting intervention. An accompanying program consisting of physical exercise, mindfulness, and meditation will be provided and group interaction will be favored. The training program includes daily outdoor walks of 1.5 h, moderate-intensity fitness exercises and free access to the gym and swimming pool.

### Outcome parameters

An overview of the main study outcomes is shown in Figure 1.

#### Body composition

**FIGURE 3:**
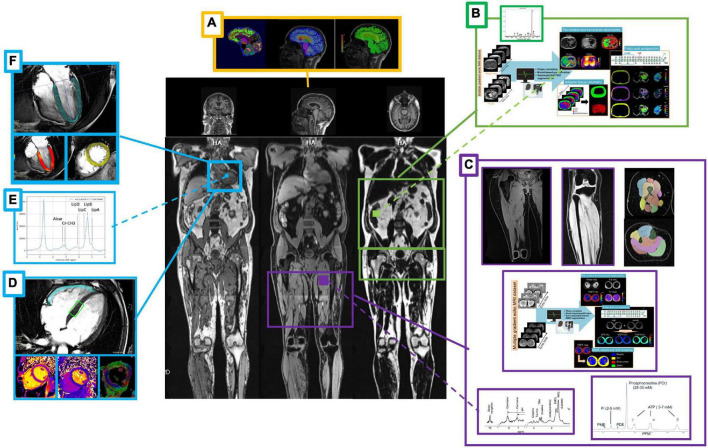
Summary of the magnetic resonance imaging/spectroscopy (MRI/MRS) measurements. Body composition after a 12 days fasting period by means of MRI/MRS scans with a focus on the brain **(A)**, liver **(B)**, skeletal muscle **(C)**, and myocardium **(D–F)**. Beyond brain morphometry **(A)**, myocardial mass, function and regional deformation **(F)**, and abdominal organs sizing **(B)** calculated from localized dedicated scans (side panels). Subcutaneous, visceral, extra-visceral and bone marrow fat quantification and total lean mass will be calculated from whole body acquisition (middle panel). MR spectroscopy of the liver **(B)**, leg muscle **(C)**, and heart **(E)** allows complementary fat decomposition, triglycerides and metabolites concentrations quantification. Advanced myocardium tissue characterization will include relaxometry and diffusion parameters mapping, as well as myocardial fiber orientation and local deformation **(D)**. Muscle legs volume and strength (maximum voluntary contraction) will be compared to MRS 31P data and extracted biomarkers of the oxidative metabolism **(C)**.

The primary endpoints in this study are changes in whole body composition (fat mass, lean mass, and water) at the end of the fasting intervention as well as 1 and 4 months afterward compared to baseline measured by MRI (44–46) (Figure 3). Furthermore, changes in size and composition of heart (47–52), liver (53, 54), kidney, spleen, adipose tissue, lumbosacral muscle mass, and lower limbs (quadriceps, hamstrings, and calves) (55) over time as well as changes in brain morphometry (56, 57) will be assessed. Details of MRI measurements are provided as Supplementary material.

Changes in bio-electrical multifrequency impedance analysis will enrich the MRI/MRS scans (58–60) with information about global and segmental body composition (water, fat, and lean mass), liquid distribution (total, extracellular, and intracellular water), metabolic indexes [metabolic activity index (MAI)], and protein content (total and active cell mass fraction). Furthermore, the CV fitness will be assessed by measuring maximal oxygen consumption (VO2max) on cycloergometers (61). Triaxial actigraphy will allow a continuous recording of the physical activity and sleep quality during the whole study. Last, we will use standardized morning heart rate variability measurements (Polar H10 with Kubios HRV mobile app.) to assess the autonomous nervous system response along the protocol (62, 63).

#### Lipid function

High-density lipoprotein, CEC, and serum CLC will be analyzed as previously described (64–66). Additional information will be gained through the analysis of chylomicrons (67), HDL and LDL subfractions, lipoprotein transfer enzymes (Cholesteryl Ester Transfer Protein activity) (68), proprotein convertase subtilisin/kexin type 9 (PCSK9) (69), paraoxonase (PON-1) activity (70), serum amyloid A levels, GlycA (71), apolipoproteins AI and B (71), lipoprotein(a) (72), and oxidized phospholipids (73). The fatty acid profile in erythrocyte membranes will provide additional insights at the molecular level (74).

#### Metabolic pathways

An analysis of the metabolome in PBMCs will be performed using combinations of untargeted and targeted LC/MS approaches (2).

#### Sulfur signaling

To address the transsulfuration pathway, sulfur compounds (amino acids, thiosulfate, and H_2_S) will be analyzed in serum and urine at baseline, after three fasting days, at the end of fasting, as well as 1 month post food reintroduction (75).

#### DNA methylation profiling

Experimental surrogate indicators of biological age [so-called epigenetic aging clocks) will be analyzed using methylation arrays from blood DNA methylation (76)].

#### Gut microbiota composition

Stool samples will be collected for shotgun metagenomics to determine the fecal microbiota composition in all participants prior to fasting and from the first spontaneous stool after the fasting period as previously described (6). We will evaluate microbial composition by identifying species-specific marker genes and functional potential by profiling microbial metabolic pathways and other molecular functions as described in other published fecal microbiome studies (77). The study of carbohydrate metabolism will be complemented by a carbohydrate-active enzyme (CAZy) profiling method that is currently in development at european molecular biology laboratory (EMBL) Heidelberg. All raw microbial data will be made available on public repositories.

#### Clinical data

Clinical data (e.g., body weight, body-mass-index, systolic and diastolic blood pressure, and heart frequency) will be documented at baseline, during fasting and food reintroduction as well as 1 and 4 months afterward. The abdominal circumference will be measured before and after fasting. Changes in ketonuria, capillary ketonemia, and capillary blood glucose levels will be measured during the fasting period and food reintroduction. Nitrogen balance, measured in 24-h urine samples (15), will be determined at baseline and during the fasting and food reintroduction period in the MRI subgroup only.

A clinical standard laboratory blood profile will be measured in each blood sample, during the transition day and after fasting (10). Additional blood samplings will be obtained from the first 32 subjects after three fasting days as well as 1 month afterward.

#### Questionnaires

Several validated questionnaires will be recorded at baseline, at the end of fasting as well as 1 and 4 months afterward to document mental wellbeing (Warwick-Edinburgh Mental WellBeing Scale) (78), global health (PROMIS Scale) (79), physical activity (Godin Leisure-Time Exercise Questionnaire) (80), sleep quality (Pittsburgh Sleep Quality Index) (81), and dietary behavior (short healthy eating index survey) (82). Lifestyle habits like smoking behavior, alcohol consumption, or physical activity in hours/week will be self-reported. Participants will document energy level, emotional and physical wellbeing, as well as symptoms including fatigue, muscle weakness, back pain, hunger, anxiety, headache, and sleep disturbances on visual scales (0–10) at baseline, daily during fasting and food reintroduction as well as 1 and 4 months afterward.

Adverse events will be documented continuously.

### Data analysis plan

The data of all clinical endpoints will be collected before and after 9 ± 3 fasting days. Demographic data and each participant’s medical history will be captured at baseline. The detailed timing of all measurements is shown in Table 2.

**TABLE 2:** Summary of measurements.

	Before	Transition	Fasting days													Food reintroduction	+1 month	+4 months
Measurements	−5 to −2	−1	0	1	2	3	4	5	6	7	8	9	10	11	12			
Demographic data	x																	
Medical history	x																	
Anthropometric measurements	x	x	x	x	x	x	x	x	x	x	x	x	x	x	x	x	x	x
Vital signs	x	x	x	x	x	x	x	x	x	x	x	x	x	x	x	x	x	x
Blood sampling		x				x			x								x	
Capillary blood sampling	x	x	x	x	x	x	x	x	x	x	x	x	x	x	x	x		
First morning urine sampling			x			x			x								x	
24 h urine sampling	x	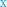	x	x	x	x	x	x	x	x	x	x	x	x		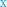		
MRI	x												x				x	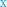
MRS P31 and H1	x												x				x	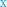
Bioelectrical impedance analysis	x												x			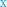	x	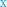
Quadriceps muscle ergometry	x												x				x	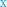
Triaxial actigraphy	x	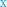	x	x	x	x	x	x	x	x	x	x	x	x		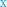	x	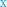
Spiroergometry	x										x						x	
Stool sampling	x															x		
Visual scores: wellbeing, symptoms	x	x	x	x	x	x	x	x	x	x	x	x	x	x	x	x	x	x
Questionnaires and life style		x											x				x	x
Adverse events	x	x	x	x	x	x	x	x	x	x	x	x	x			x	x	x

Measurements only conducted in the first stage of this study (*n* = 32) are highlighted in blue.

Further, the Supplementary File contains a detailed description of the anthropometric data, vital signs, blood, urine and stool samples, the MRI and MRS acquisition protocols, bioelectrical impedance analysis, quadriceps muscle ergometry, triaxial actigraphy, and spiroergometry.

### Sample size calculation

The required sample sizes vary between the outcome parameters. Previously, the effect size in matched subjects was 0.81 for T2 relaxometry in muscles (55). Using these assumptions, the minimal sample size requirement is 14 to detect meaningful acute changes in muscle physiology (alpha = 0.05, power = 0.80). MRS studies monitoring triglyceride (TG) content during CR (83, 84) observed that effect sizes were 1.09 and 0.59 in liver and muscle, respectively. Using the small effect size of muscle changes (alpha = 0.05, power = 0.80), the required sample size to detect organ-specific differences in MRS measurements is 28. To account for a potential drop-out rate of 15%, the sample size was set to 32.

Clinical routine laboratory examinations and other blood sample analyses will be performed on 100 samples. This number of participants was chosen to reach sufficient power for detecting differences in gut metagenome analysis. Large inter-individual differences are usually observed in the composition of fecal microbiomes. Small differences in alpha diversity (effect size 0.55) between two groups of 50 individuals can be detected with an 80% statistical power (85). Missing values are commonly encountered in fecal microbiome evaluations (85). Accounting for missing data and sufficient numbers to quantify bacteria abundance at the species level for at least 50 individuals, we estimated that 100 subjects are needed for this part of the study (power = 0.80). This is based on a fecal metagenome analysis, which included missing values for more than 50% of 612 bacterial species out of 724 species detected (86).

### Data collection and management

All research data will be documented on case report forms and the study diary. A pseudonymised identification number will be allocated to each participant. Study data will be transcribed from the source documents and stored electronically in a pseudonymised, password-secured database at Buchinger Wilhelmi Development und Holding GmbH. Documents allowing the identification of subjects (e.g., signed consent forms) will be stored securely and separately from the study data. Only representatives of the principal investigator, who are obliged to maintain confidentiality, have access to these data. The follow-up data will be collected using an online questionnaire that meets general data protection regulation standards. Data will always be treated confidentially and data protection regulations will be complied with. The source data will be kept for at least 10 years after the termination of the study.

### Statistical analysis

The statistical analysis of the data will be performed using R software for statistical computing. Given the longitudinal design of our study with multiple repeated measurements, a mixed model for repeated measures will be used for quantitative variables. The *p*-values from linear-mixed models in the longitudinal data analysis will be adjusted with a *post-hoc* Tukey test. Missing data will be handled with complete case analyses for the dependent variables or imputed using the median for auxiliary variables with less than 20% missing values. Subgroups will be generated using demographic data (e.g., sex) or unsupervised clustering methods. The statistical models will be adjusted for potential confounders such as age, gender, or baseline body weights. A parsimonious approach will be used. If a covariate does not influence the variance of the model, it will be excluded from the model. We will use multivariate statistics for metabolomics or metagenomics, including unsupervised (e.g., principal component analysis) and supervised statistical methods (orthogonal partial least squares discriminant analysis, sparse partial least square discriminant analysis). In the case of multivariate analyses (e.g., microbiome data), the false discovery rate corrected *p*-value (*q*-value of 5%).

## Ethics and dissemination

The ethics commission of the federal state of Baden-Württemberg approved the study protocol on 26th July 2021. It was registered in an official clinical trial register on 2nd September 2021 (ClinicalTrials.gov Identifier: NCT05031598). The ethics commission will approve all protocol amendments and update the clinical trial register. The study will be conducted in accordance with the Declaration of Helsinki and the guidelines of the International Conference of Harmonization of Good Clinical Practice Guidelines and German law.

Microbiota data will be made public in a data depository. Results will be published in peer-reviewed journals and presented at international conferences and on social media.

## Data availability statement

The original contributions presented in the study are included in the article/Supplementary material, further inquiries can be directed to the corresponding author.

## Ethics statement

The studies involving human participants were reviewed and approved by Ethics Board of the State Medical Chamber of Baden-Württemberg. The patients/participants provided their written informed consent to participate in this study.

## Author contributions

FG: project administration, methodology, data collection, and writing—original draft preparation. MV and PC: conceptualization, funding, methodology, data collection, data analysis, and writing—review and editing. RM and SJH: methodology, writing—review and editing. MR, CvS, SJM, and FM: methodology. FWT: conceptualization, funding acquisition, supervision, and writing—review and editing. All authors contributed to the article and approved the submitted version.
