# Supplementary material for: Long-term fasting: Multi-system adaptations in humans (GENESIS) study–A single-arm interventional trial
Source: Front Nutr. 2022 Nov 17;9:951000. doi: 10.3389/fnut.2022.951000 (PMC9713250; doi:10.3389/fnut.2022.951000)
Supplement: Supplementary file 1 [file Data_Sheet_1.docx]

Supplementary Material

Detailed description of the measurements

**Long-term fasting: multi-system adaptations in humans (GENESIS) study - a single-arm interventional trial**

**Franziska Grundler (1)*, Magalie Viallon (2,3)*, Robin Mesnage (1,4), Massimiliano Ruscica (5), Clemens von Schacky (6), Frank Madeo (7,8,9), Sebastian J. Hofer (7,8,9), Sarah J. Mitchell (10), Pierre Croisille (2,3)†, Françoise Wilhelmi de Toledo (1)†**

^1^Buchinger Wilhelmi Clinic, Wilhelmi-Beck-Straße 27, 88662 Überlingen, Germany

^2^Univ Lyon, UJM-Saint-Etienne, INSA, CNRS UMR 5520, INSERM U1206, CREATIS, F-42023, Saint-Etienne, France

^3^Department of Radiology, University Hospital Saint-Etienne, Saint-Etienne, France

^4^King’s College London, Faculty of Life Sciences & Medicine, Department of Medical and Molecular Genetics, 8th Floor, Tower Wing, Guy’s Hospital, Great Maze Pond, London, SE1 9RT, United Kingdom

^5^Department of Pharmacological and Biomolecular Sciences, Università degli Studi di Milano, Milan, Italy

^6^Omegametrix, Am Klopferspitz 19, 82152 Martinsried, Germany

^7^Institute of Molecular Biosciences, NAWI Graz, University of Graz, Graz Austria

^8^BioHealth Graz, Graz Austria

^9^BioTechMed Graz, Graz Austria

^10^Department of Health Sciences and Technology, ETH Zürich, Schorenstrasse 16, 8603 Schwerzenbach, Switzerland

*****These authors have contributed equally to this work and share first authorship.

**†** These authors have contributed equally to this work and share last authorship.

## Anthropometric data and vital signs

Height will be measured at the beginning of the study, prior to fasting, by a physician. Body weight will be recorded continuously by trained personnel and the participants will wear light clothing without shoes (seca 704, seca, Hamburg, Germany). Blood pressure and heart frequency will be measured during the fasting stay once at the non-dominant arm in a sitting position after a resting period (BOSCH + SOHN GmbH u. Co. KG, Jungingen, Germany). Additionally, the mean blood pressure and heart frequency will be determined before and after the MRI scans. Abdominal circumference will be measured before and at the end of fasting, using a tape placed horizontally, midway between the lowest rib and iliac crest.

## Blood, urine and stool samples

Blood will be sampled by trained medical-technical assistants between 7:30 am and 9:30 am at baseline and the end of fasting. We will sample additional blood samplings of the MRI sub-group after three fasting days as well as one month afterward. The blood samples will be either directly transported to a fully automated laboratory for analysis of routine parameters or further processed and frozen at -70°C. Serum samples will be collected in tubes including gel with a clotting activator (S-Monovette 7 mL Z-Gel, Sarstedt, Nümbrecht, Germany) and centrifuged for 10 min at 5000 rpm. The measurements will include leucocytes, erythrocytes, haemoglobin, haematocrit, mean corpuscular volume (MCV), mean corpuscular haemoglobin (MCH), mean corpuscular haemoglobin concentration (MCHC), thrombocytes, bilirubin, basophils, neutrophils, eosinophils, monocytes, reticulocytes), lipids (total cholesterol, triglycerides (TG), LDL-C, HDL-C), glucose parameters (glucose, glycated haemoglobin (HbA1c), insulin), liver parameters (glutamic oxaloacetic transaminase (GOT), glutamate pyruvate transaminase (GPT), gamma-glutamyl transferase (GGT), alkaline phosphatase (AP)), kidney (uric acid, urea, creatinine) and inflammatory parameters (high-sensitivity c-reactive protein (hs-CRP), erythrocyte sedimentation rate (ESR), cortisol) as well as coagulation (international normalized ratio (INR), partial thromboplastin time (PTT)) and electrolytes (sodium, potassium, calcium, magnesium. EDTA samples (S-Monovette 2.7 mL K3 EDTA, Sarstedt, Nümbrecht, Germany) will be sent to the laboratory in Munich, fresh and deep frozen.

Capillary blood samples will be obtained by a fingertip prick with a lancet. Special test strips for glucose and ketonemia levels will be evaluated using GlucoMen areo 2K (Berlin-Chemie AG, Berlin, Germany).

In parallel to the blood samples, midstream urine samples will be collected, starting the first morning, and stored deep-frozen. Additionally, a semiquantitative analysis using urine dipsticks (Ketostix, Bayer AG) will be performed daily throughout the study. In the MRI subgroup, 24-hour urine will be collected continuously from baseline until day four of the food reintroduction phase.

Stool samples will be collected twice, at baseline and from the first spontaneous stool after fasting, with a stool collection kit (EasySampler, GP Medical Devices A/S, Holstebro, Denmark). Stoll samples will be stabilized with a DNA/RNA Shield (Zymo Research Europe GmbH, Freiburg, Germany) and stored at -70°C.

## Serum HDL fraction

HDL cholesterol efflux capacity (HDL CEC) will be evaluated on the HDL fraction isolated from whole serum by precipitating the apoB-containing lipoproteins with polyethylene glycol (1). To avoid lipoprotein remodelling, sera will be slowly defrosted in ice immediately before this procedure. HDL CEC occurring through the main cholesterol efflux pathways will be evaluated by a standardized and widely used radioisotopic cell-based technique as previously described (1, 2). Whole serum cholesterol loading capacity (CLC) will be evaluated by fluorometry in human monocyte-derived macrophages THP-1 (1). Serum Amyloid A in the isolated HDL serum fraction will be quantified by colorimetric ELISA assays (Human Serum Amyloid A ELISA KIT—Sigma Aldrich), following the manufacturer’s instructions, with a minimum detection range of 500 pg/mL (3). HDL-bound PON-1 activity will be measured in the isolated HDL serum fraction using the commercially available fluorometric PON-1 Activity Assay Kit (BioVision, Milpitas, CA, USA), according to the manufacturer’s guidelines (3, 4).

## Erythrocyte fatty acid composition

Erythrocyte fatty acid composition will be analysed according to the HS-Omega-3 Index® methodology as previously described (5). Fatty acid methyl esters were generated from erythrocytes by acid transesterification and analysed by gas chromatography using a GC2010 Gas Chromatograph (Shimadzu, Duisburg, Germany) equipped with an SP2560, 100-m column (Supelco, Bellefonte, PA) using hydrogen as carrier gas. Fatty acids were identified by comparison with a standard mixture of fatty acids characteristic of erythrocytes. Results are given as EPA plus DHA expressed as a percentage of total identified fatty acids after response factor correction. The coefficient of variation for EPA plus DHA was 5%. Analyses were quality-controlled according to DIN ISO 15189.

## PBMC isolation

PBMCs will be isolated from freshly drawn lithium-heparin blood (S-Monovette, 9 mL, lithium-heparin, Sarstedt, Nümbrecht, Germany) according to the manufacturer’s instructions (Stemcell Technologies, Cologne, Germany) using SepMate^TM^ PBMC Isolation Tubes.

## Transsulfuration pathway

Persulfidation will be measured using our recently published method (6). Hydrogen sulphide production capacity will be determined using the lead acetate assay (7, 8). Serum and urine levels of selected sulphur compounds will be measured in response to fasting. Specifically, thiosulfate will be measured using the monobromobimane method using high performance liquid chromatography (HPLC) and liquid chromatography-tandem mass spectrometry (LC-MS/MS) (9). We will also use the S-sulfocysteine (SSC) method which is HPLC-based, using automated precolumn derivatization with OPA and UV detection at 338 nm to measure cysteine and cystine in serum (10). Finally, we will measure amino acid metabolites in the serum and urine using a targeted approach.

## Faecal microbiota

DNA will be extracted from stabilized stool samples using the Quick-DNA Fecal/Soil Microbe Miniprep Kit (ZymoResearch) with minor adaptations of the manufacturer’s instructions as previously described (11). Shotgun metagenomics will be performed by fragmenting, A-tailing, and ligating sequencing adapters to the resulting product. Quality and yield after sample preparation will be documented using a fragment analyser. Clustering and DNA sequencing will be performed on NovaSeq6000 to generate 5-10 Gb per sample. Shotgun metagenomics data sets will be pre-processed before pathway and community profiling to remove adapters, trim low-quality positions and unknown positions, and discard low-quality (i.e., quality <20 or >2 Ns) or too-short reads (<75 bp), removing contaminants (phiX and human genome sequences).

## MRI and MRS acquisition protocol

The MRI and MRS scans will be acquired with a clinical 3.0 Tesla MR Imaging system (*Magnetom* Prisma, Siemens *HealthCare*, Erlangen, Germany). The MR explorations will cover brain morphometry, body composition from neck to knee examination for total, lean and fat mass, organ size and tissue characterization using relaxometry and diffusion with a special focus on major organs such as the brain, heart, liver, and skeletal muscle. In particular, it will involve advanced techniques to evaluate the regional and global heart function, including strain analysis (12) up to Myofiber strain (Eff), a surrogate of cardiomyocyte contraction (13). Indeed, a major focus will be put on the study of metabolism and muscle function. E_ff_ is an essential and unique functional and microstructural biomarker of cardiac tissue. E_ff_ represents, at the voxel scale, the percentage of contraction that undergoes the cardiomyocytes and thus is used to estimate their performance and wellness. Single-voxel 1H magnetic resonance spectroscopy (1H-MRS) will complete the analysis in the liver, heart and quadriceps to quantify intramyocardial lipids and metabolites, such as creatine (Cr), the variations of myocardial triglyceride (TG) metabolism, the intra- and extra-myocellular lipids. Finally, dynamic 31-MRS will be performed to complete this in-depth study of muscle metabolism, muscle energy and muscle fatigability (14). 31P-MRS during exercise allows the assessment of the concentration of phosphorylated metabolites, which are directly related to the respiratory capacity of the mitochondria (15, 16).

The total imaging time for the 1H protocol is approximately 90 min, including coil changes and the switch to different anatomical parts. The total imaging time for the dual 31P & 1H protocol is about 35 min, including the dynamic exercise involving plantar flexions using an MR-compatible ergometer.

## Brain imaging

Brain morphometry will be assessed using the MorphoBox algorithm (developed by the Advanced Clinical Imaging Technology group in Lausanne, Siemens Healthcare) that automatically estimates a number of brain sub-volumes from a single T1-weighted MR image acquired with the MPRAGE sequence prototype sequence (17). The results are presented to the user as a DICOM report. The software compares brain volumes with normative ranges adjusted for head size, age, and sex (18).

## Body composition

A multi-step neck-to-knee protocol will be implemented using axial 3D spoiled gradient dual-echo images divided over six overlapping slabs of axial 3D spoiled gradient dual-echo images. Over the torso region (slabs two to four), imaging will be performed during 19 s expiration breath-hold. The total time for the MR body composition protocol will be about 6 minutes. Dixon water and Dixon fat images will be reconstructed in-line using the integrated scanner software. Body composition profiling of all participants will be obtained by quantifying fat and muscle compartments such as visceral adipose tissue, abdominal subcutaneous adipose tissue, and thigh muscle volumes, as well as intra-muscular and liver fat content (19-21).

## Fat volume and profile

Chemical Shift-Encoded Magnetic Resonance Imaging (CS-MRI) acquisitions will be sequentially performed for simultaneous quantification of fat content and T2∗ in the abdomen, tight muscles and femur bone marrow, as well as in the spine and hip bone marrows. Bone marrow (BMAT), visceral (VAT) and subcutaneous (SAT) adipose tissues will be quantified simultaneously. At the same time, the fatty acid composition will be obtained as previously described (22, 23) using a fat 1H MR spectrum model integrating eight components. The number of double bonds (ndb) and methylene-interrupted double bonds (nmidb) will be derived voxel by voxel by a step-wise data fitting procedure on the corrected signal.

MR spectroscopy (1H-MRS) of the liver, quadriceps muscle and myocardium will provide a robust analysis of organs with less than 3% fat, for which multi-echo MRI lacks sensitivity and robustness. Single-voxel liver 1H-MRS data in the liver will be acquired using a point-resolved spatially localized spectroscopy pulse sequence with and without water suppression. Metabolite quantification will be performed using the QUEST (QUantitation based on QUantum ESTimation) method within MatLab (The MathWorks) (24). A customized fitting algorithm will be used for lipid signals at 0.9, 1.3, 1.6, 2.3, 5.2 and 5.3 ppm. Olefinic protons at 5.2 and 5.3 ppm and methylene protons at 1.3 ppm will be used as estimates of fatty acid unsaturated bonds and fatty acid saturated bonds, respectively. The saturation index (SI) will be determined by obtaining a ratio between the methylene protons at 1.3 ppm and total lipid content. Unsaturated lipid estimates will be obtained from water-suppressed spectra and the unsaturation index (UI) from a ratio of the olefinic resonance at 5.2 and 5.3 ppm and total lipid content.

Single-voxel 1H cardiovascular magnetic resonance spectroscopy (1H-CMRS) will be performed to quantify intramyocardial lipids and metabolites, such as creatine (Cr), and the variations of myocardial triglyceride (TG) metabolism. The CMRS acquisition voxel size will be placed in the interventricular septum, single-voxel CMRS will be performed using conventional point resolved spectroscopy (PRESS) sequence and semi‑adiabatic Localization by Adiabatic SElective Refocusing (sLASERPRESS, sLASER provided by Center for Magnetic Resonance Research (CMRR, VB17, available at https:// www. cmrr. umn. edu/ spect ro/). The sequence includes suppression of water (W) signals using variable pulse power and optimized relaxation delays (VAPOR) (25).

CMRS will be quantified with a time-domain CMRS model consisting of a linear combination of numerically computed metabolite spectra with a homemade Python software (26). The pyGAMMA simulation library (27) will be employed to simulate tCr (3.027 ppm), myocardial TG and TMA (3.183 ppm) using a spin-echo acquisition. Myocardial TG will be modeled using 6 Gaussian components in total [8] and two groups: FA at 0.9, 1.3 and 1.6 ppm and unsaturated FA (UFA) at 2.1, 2.3 and 2.8 ppm. The CMRS model will be numerically adjusted to the data using a non-linear least-squares optimization algorithm resulting in relative concentration and frequency shift estimates for each metabolite as well as an overall linewidth damping and phase shift. Cramér-Rao Lower Bounds (CRLB) will be estimated considering a noise level measured on the unprocessed, unfiltered raw data. The ratios FA/W and Cr/W will be provided as percentages after T1 and T2 correction.

## Cardiac structuro-functional imaging

The CMR protocol includes a multi-level short-axis, 2- and 4-chamber views, Left ventricular Outflow track cine bSFFP sequence for left ventricle (LV) function analysis. LV mass, LV and RV functional parameters such as left-ventricular ejection fraction (LVEF), peak global longitudinal strain (GLS), peak global radial strain (GRS) and peak global circumferential strain (GCS) will be derived from the short-axis cine images using a commercially available software package (CMR42, Circle).

Mid-ventricular short-axis parametric maps will be acquired using a MOLLI pulse sequence (28) for T1 maps and a T2-prepared bSSFP (29). Cardiac Diffusion Weighted Imaging (cDWI) will be acquired using a single shot spin-echo echo-planar imaging (SE-EPI) using M12 motion compensated diffusion encoding (30). Five double-oblique short-axis DW image slices of 6 mm thickness will be obtained in systole. Twelve diffusion directions will be acquired with a main b-value of 350s/mm² and a reference b-value of 5s/mm². After DWI pre-processing and image registration, ADC, FA and HA maps will be calculated offline in Matlab (The MathWorks Inc, Natick, MA). Myofiber strain (E_ff_) is a surrogate of cardiomyocyte contraction will be calculated as previously described (13). E_ff_ is essential and unique bio-mechanistically as it is both a functional and microstructural biomarker of the cardiac tissue. E_ff_ represents, at the voxel scale, the percentage of contraction that cardiomyocytes undergo and thus could be directly used to estimate performance. The total imaging time for the cardiac protocol will require approximately 45 min.

## 31-MRS and Muscle metabolism

Dynamic 31P-MRS acquisition will be performed using an MR-compatible ergometer to evaluate skeletal muscle energy and muscle fatigability (14). 31P-MRS during exercise allows the assessment of phosphorylated metabolite concentrations, which is directly related to the respiratory capacity of mitochondria (15, 16).

The data will be processed with MatLab (The MathWorks) and the QUEST method in the command line version, using a metabolite basis set consisting of phosphocreatine (PCr), inorganic phosphate (Pi), α-, β-, and γ-Adenosine Tri Phosphate (ATP) as a priori knowledge to estimate the amplitude, frequency, and phase parameters of each metabolite. The metabolite base was obtained by adjusting Lorentzian line shapes at the resonance positions of distinct signals related to PCr for PCr (0ppm), Pi (5.02ppm), ATPbeta (-16.26ppm) and a doublet for gamma (-2.48ppm) and alpha (-7.52ppm) ATP. The concentration of adenosine diphosphate (ADP) can be calculated as a function of pH and [PCr], assuming creatine kinase (CK) equilibrium. A single-exponential fit will be performed on the exercise and recovery periods of PCr and Pi amplitudes, to extract the time constants of PCr (τPCrEx and τPCrRec) and Pi (τPiEx and τPiRec). From these data, we will calculate the initial recovery rate of PCr (ViPCr) and the maximum aerobic capacity (Vmax). This model also permits extracting Vmax from the resting concentration of PCr. Furthermore, the ViPCr allows us to access the contractile cost generated by muscular effort. Finally, we will calculate the PCr/Pi ratio at rest, which strongly indicates oxidation states.

## Bioelectrical impedance analysis

Global and segmental body composition (water, fat and lean mass), liquid distribution (total extracellular and intracellular water), metabolic indexes (metabolic activity index) and protein content (total and active cell mass fraction) will be assessed using a bioelectrical multifrequency impedance analysis (Z-Métrix, Bioparhom, Challes Les Eaux, France).

## Quadriceps muscle ergometry

Patients will be seated on an isometric dynamometer (ARS dynamometry; SP2, Ltd., Ljubljana, Slovenia) with a knee and hip angle set at 90°. Maximum voluntary contraction (MVC) measurements will be performed by default on the right side. Extraneous movements of the body will be minimized by a strap across the abdomen and instructions to keep the hands on the thorax. After a standardized warm-up consisting of submaximal contractions, the participants will perform three MVCs separated by 60 s.

## Triaxial actigraphy

Accelerometer-based measurements will be performed using a wrist-worn triaxial actigraph (CamNtech Ltd., Cambridgeshire, UK). Physical activity monitoring (intensity, duration, frequency), circadian rhythm analysis and sleep analysis will be conducted one week before the fasting period, during fasting, as well as up to one month afterward.

## Spiroergometry

The spiroergometry test will be performed on a bicycle ergometer and supervised by a trained physician. The lung function will be documented at rest by spirometry. The equipment of the respiratory gas analysis system will be calibrated before each test. Heart frequency will be measured by a chest belt and a 12-lead electrocardiography will be conducted. The participants will wear a face mask to collect respiratory data. The spiroergometry will be performed as a ramp test with increasing performance (W) and a target duration of 10 min. The potential termination value is defined by the individual result of a physical working capacity test with a target heart frequency of 150 beats/minute. Before the measurement, the participants will be verbally encouraged to cycle on the ergometer until exhaustion. The protocol will start with 2-minute cycling without load as a warm-up. A full set of spiroergometric parameters will be documented, including minute ventilation (VE), breathing frequency, oxygen uptake (VO2) and carbon dioxide production (VCO2), heart rate and derived parameters such as respiratory exchange rate (31). The test will be terminated if the subjects cannot perform more than 60 revolutions per minute.

REFERENCES:

1. Adorni MP, Zimetti F, Cangiano B, Vezzoli V, Bernini F, Caruso D, et al. High-density lipoprotein function is reduced in patients affected by genetic or idiopathic hypogonadism. J Clin Endocrinol Metab. 2019;104(8):3097-107.

2. Greco D, Kocyigit D, Adorni MP, Marchi C, Ronda N, Bernini F, et al. Vitamin D replacement ameliorates serum lipoprotein functions, adipokine profile and subclinical atherosclerosis in pre-menopausal women. Nutrition, Metabolism and Cardiovascular Diseases. 2018;28(8):822-9.

3. Papotti B, Macchi C, Favero C, Iodice S, Adorni MP, Zimetti F, et al. HDL in COVID-19 Patients: Evidence from an Italian Cross-Sectional Study. Journal of Clinical Medicine. 2021;10(24):5955.

4. Mohammed CJ, Xie Y, Brewster PS, Ghosh S, Dube P, Sarsour T, et al. Circulating Lactonase Activity but Not Protein Level of PON-1 Predicts Adverse Outcomes in Subjects with Chronic Kidney Disease. Journal of Clinical Medicine. 2019;8(7):1034.

5. Köhler A, Bittner D, Löw A, von Schacky C. Effects of a convenience drink fortified with n-3 fatty acids on the n-3 index. Br J Nutr. 2010;104(5):729-36.

6. Zivanovic J, Kouroussis E, Kohl JB, Adhikari B, Bursac B, Schott-Roux S, et al. Selective Persulfide Detection Reveals Evolutionarily Conserved Antiaging Effects of S-Sulfhydration. Cell Metab. 2020;31(1):207.

7. Hine C, Harputlugil E, Zhang Y, Ruckenstuhl C, Lee BC, Brace L, et al. Endogenous hydrogen sulfide production is essential for dietary restriction benefits. Cell. 2015;160(1-2):132-44.

8. Hine C, Mitchell JR. Endpoint or Kinetic Measurement of Hydrogen Sulfide Production Capacity in Tissue Extracts. Bio Protoc. 2017;7(13).

9. Kožich V, Ditrói T, Sokolová J, Křížková M, Krijt J, Ješina P, et al. Metabolism of sulfur compounds in homocystinurias. Br J Pharmacol. 2019;176(4):594-606.

10. Belaidi AA, Arjune S, Santamaria-Araujo JA, Sass JO, Schwarz G. Molybdenum cofactor deficiency: a new HPLC method for fast quantification of s-sulfocysteine in urine and serum. JIMD Rep. 2012;5:35-43.

11. Mesnage R, Teixeira M, Mandrioli D, Falcioni L, Ducarmon QR, Zwittink RD, et al. Use of shotgun metagenomics and metabolomics to evaluate the impact of glyphosate or Roundup MON 52276 on the gut microbiota and serum metabolome of Sprague-Dawley rats. Environ Health Perspect. 2021;129(1):017005.

12. Zweerink A, van Everdingen WM, Nijveldt R, Salden OAE, Meine M, Maass AH, et al. Strain imaging to predict response to cardiac resynchronization therapy: a systematic comparison of strain parameters using multiple imaging techniques. ESC Heart Failure. 2018;5(6):1130-40.

13. Moulin K, Croisille P, Viallon M, Verzhbinsky IA, Perotti LE, Ennis DB. Myofiber strain in healthy humans using DENSE and cDTI. Magn Reson Med. 2021;86(1):277-92.

14. Karkouri J, Slade J, Ratiney H, Grange S, Tonson A, Croisille P, et al., editors. 31P MRS assessments of mitochondrial dysfunction in patients with peripheral arterial disease undergoing revascularization. International Society of Magnetic Resonance in Medicine; 2020.

15. Kemp GJ, Meyerspeer M, Moser E. Absolute quantification of phosphorus metabolite concentrations in human muscle in vivo by 31P MRS: a quantitative review. NMR in Biomedicine: An International Journal Devoted to the Development and Application of Magnetic Resonance in Vivo. 2007;20(6):555-65.

16. Meyerspeer M, Boesch C, Cameron D, Dezortová M, Forbes SC, Heerschap A, et al. 31P magnetic resonance spectroscopy in skeletal muscle: Experts' consensus recommendations. NMR Biomed. 2021;34(5):e4246.

17. Mietchen D, Gaser C. Computational morphometry for detecting changes in brain structure due to development, aging, learning, disease and evolution. Front Neuroinform. 2009;3:25.

18. Mortamet B, Bernstein MA, Jack Jr CR, Gunter JL, Ward C, Britson PJ, et al. Automatic quality assessment in structural brain magnetic resonance imaging. Magnetic Resonance in Medicine: An Official Journal of the International Society for Magnetic Resonance in Medicine. 2009;62(2):365-72.

19. West J, Dahlqvist Leinhard O, Romu T, Collins R, Garratt S, Bell JD, et al. Feasibility of MR-based body composition analysis in large scale population studies. PLoS One. 2016;11(9):e0163332.

20. Borga M, Thomas EL, Romu T, Rosander J, Fitzpatrick J, Dahlqvist Leinhard O, et al. Validation of a fast method for quantification of intra‐abdominal and subcutaneous adipose tissue for large‐scale human studies. NMR Biomed. 2015;28(12):1747-53.

21. Middleton MS, Haufe W, Hooker J, Borga M, Dahlqvist Leinhard O, Romu T, et al. Quantifying abdominal adipose tissue and thigh muscle volume and hepatic proton density fat fraction: repeatability and accuracy of an MR imaging–based, semiautomated analysis method. Radiology. 2017;283(2):438-49.

22. Leporq B, Lambert SA, Ronot M, Vilgrain V, Van Beers BE. Quantification of the triglyceride fatty acid composition with 3.0 T MRI. NMR Biomed. 2014;27(10):1211-21.

23. Viallon M, Leporq B, Drinda S, Wilhelmi de Toledo F, Galusca B, Ratiney H, et al. Chemical-Shift-Encoded Magnetic Resonance Imaging and Spectroscopy to reveal immediate and long-term multi-organs composition changes of a 14-days periodic fasting intervention: a technological and case report. Frontiers in Nutrition. 2019;6:5.

24. Ratiney H. Quantification automatique de signaux de spectrométrie et d'imagerie spectroscopique de résonance magnétique fondée sur une base de métabolites: une approche semi-paramétrique: Lyon 1; 2004.

25. Sourdon J, Roussel T, Costes C, Viout P, Guye M, Ranjeva J-P, et al. Comparison of single-voxel 1H-cardiovascular magnetic resonance spectroscopy techniques for in vivo measurement of myocardial creatine and triglycerides at 3T. J Cardiovasc Magn Reson. 2021;23(1):1-13.

26. Roussel T, Le Fur Y, Ranjeva J-P, Callot V, editors. Respiratory‑triggered quantitative MR spectroscopy of the human spinal cord at 7 T. . ISMRM Annual Meeting; 2020.

27. Smith S, Levante T, Meier BH, Ernst RR. Computer simulations in magnetic resonance. An object-oriented programming approach. Journal of Magnetic Resonance, Series A. 1994;106(1):75-105.

28. Messroghli DR, Radjenovic A, Kozerke S, Higgins DM, Sivananthan MU, Ridgway JP. Modified Look-Locker inversion recovery (MOLLI) for high-resolution T1 mapping of the heart. Magn Reson Med. 2004;52(1):141-6.

29. Giri S, Chung YC, Merchant A, Mihai G, Rajagopalan S, Raman SV, et al. T2 quantification for improved detection of myocardial edema. J Cardiovasc Magn Reson. 2009;11(1):56.

30. Moulin K, Viallon M, Romero W, Chazot A, Mewton N, Isaaz K, et al. MRI of reperfused acute myocardial infarction edema: ADC quantification versus T1 and T2 mapping. Radiology. 2020;295(3):542-9.

31. Brownstein CG, Espeit L, Royer N, Ansdell P, Škarabot J, Souron R, et al. Reductions in motoneuron excitability during sustained isometric contractions are dependent on stimulus and contraction intensity. J Neurophysiol. 2021;125(5):1636-46.
